# Stem Cell Activation in Adult Organisms

**DOI:** 10.3390/ijms17071005

**Published:** 2016-06-24

**Authors:** Wilhelm Bloch

**Affiliations:** Institute of Cardiovascular Research and Sport Medicine, Department of Molecular and Cellular Sport Medicine, German Sport University Cologne, Am Sportpark Muengersdorf 6, Cologne 50933, Germany; w.bloch@dshs-koeln.de; Tel.: +49-0-221-4982-5380

Stem cells are responsible for the organ and tissue development, growth and maintenance from embryonic stage up to late adult life. The potential of stem cell-based tissue regeneration and growth in the adult organism is recognized for nearly all organs and tissues, such as muscle, heart, brain, lung, skin, mesenchymal, epithelial, and connective tissues. This stem cell based regeneration potential should be preserved over the lifespan to improve health, but it is regularly impaired during aging by loss of stem cell activity. Therefore, it is important to develop strategies for the maintenance and activation of the stem cells, in order to provide protection against diseases and in the reduction of the ageing process. The comprehension of the activation mechanisms and cellular signaling induced by local and systemic factors, such as cytokines, metabolic factors, extracellular matrix, and supporting cells, allows for the improvement of these mobilization strategies. Except for the direct potential of stem cells to form specified differentiated cells in organs and tissues, its indirect effects should not be underestimated. Especially for adult mesenchymal stem cells, the protective function for other cell types under specific disease conditions, such as heart failure and inflammation, is remarkable.

In the Special Issue “Stem Cell Activation in Adult Organisms”, a promising insight into different aspects of stem cell biology is given with a specific focus to mesenchymal stem cells. In addition, dermal, ependymal, lung and tumor stem cells are addressed. For all these stem cells, the direction of the self-renewal and differentiation potency is essential. In addition to the growth factors and extracellular matrix cues, various metabolic pathways definitively provide important signals for the self-renewal and differentiation potency of stem cells. This is not surprising, if the permanent acute and chronic metabolic variances are taken into consideration. The critical role of metabolism for stem cells is described by Hu et al. [1] as a change of mitochondria from cristae poor to a cristae rich phenotype associated with a switch from glycolytic metabolism to oxidative metabolism is observed, when stem cell terminally differentiate. The metabolism will facilitate the optimization of in vitro maintenance and differentiation protocols by adjusting biochemical properties for regenerative medicine. Such a complex change of the cellular metabolism with alteration of the expression of more oxidative mitochondrial enzymes cannot be understood without changes on the functional genome by epigenetic mechanisms, such as DNA methylation and histone modification.

These epigenetic changes consist the key factor for regulation of proliferation and differentiation. The role of epigenetics is to determine the fate of stem cells and to assess how this information can be used to enhance stem cell based treatment strategies of different diseases, such as neurodegenerative disorders [2]. The current understanding of the molecular mechanisms involved in epigenetic control of stem cell differentiation into various cell lineages under specific respect of the therapeutic options for various neurological diseases is given by Srinageshwar et al. [2]. A further epigenetic mechanism for differentiation of mesenchymal stem cells to neuronal progenitor cells involves microRNAs. The study of Huat et al. [3] deciphers the differentially and uniquely expressed microRNAs involved in the differentiation of bone marrow derived mesenchymal stem cells (BMSCs) into neural lineages by microRNA profiling in BMSC-derived neuronal progenitor cells (NPCs) under the influence of insulin growth factor-1 (IGF-1). More generally, this work elucidates functions of microRNAs in stem cell differentiation, which can be at least partially used to understand the role of microRNAs in terminal differentiation of stem cells. The increased knowledge of epigenetic stem cell regulation by endogenous factors such as hormones could be used to develop strategies for maintenance and activation of stem cells in the adult organism.

The protection of stem cell fate and activation of stem cells are also derived by the local stem cell environment. Understanding the cellular cross talk between the niche and the stem cells brings out multiple options to improve stem cell dependent regenerative processes. An example for niche–stem cell interaction is given by Yoshida et al. [4]. They review the role of niches of the adult rodent pituitary by focusing on three components: Soluble factors, cell surface proteins, and extracellular matrices. The behavior of stem cells is mediated via niche cells/stem cells interaction and via ECM-to-stem/progenitor cell interactions and recruitment of soluble factors. Isolation of niches and analyses of gene expression profile, may help to understand, “How do stem/progenitor cells respond to physiological demand?”, allowing to develop strategies for regulation of the stem cell fate.

In addition to the given inside in specific stem cell fate regulating mechanisms, the special issue addresses the potential of the adult stem cells to differentiate in specific cell types to reveal regenerative capacity in the adult organisms necessary for tissue maintenance by several examples. Often focused adult stem cells are the mesenchymal stem cells (MSCs), which are located at different sides in the adult organism besides the well-known bone-marrow location. A multitude of terminal differentiation options and partially the underlying mechanisms are deciphered in the last twenty years explaining the prominent role of the MSCs during adult life. Bone formation is one of the well known potential of MSCs. Recognition of the “Secreted Frizzled-Related Protein” (sFRP-3) as activator of osteogenic MSC differentiation by Katagiri et al. [5] reveals sFRP-3 as a possible novel therapeutic agent for bone regeneration useable, e.g., as agent for the treatment of bone defects. Beside of bone development, other connective tissues are produced by terminal differentiation of MSCs, such as nucleus pulposus. Identification of factors driving the differentiation to nucleus pulposus like cells can have high relevance for maintenance of nucleus pulposus. Zhou et al. [6] demonstrated that BMP3 enhances MSC proliferation and differentiation also in concert with TGF-β helping to understand better this specific activation of MSCs. The multiple differentiation potential of MSCs together with more indirect effects which expanded the benefit of these stem cells can be used for a wide range of therapeutic approaches. One of these approaches is the osteoarthritis (OA), where MSCs can be used to replace the tissue by direct differentiation of MSCs to cell types of the joint. In addition to the differentiation of MSCs and other stem cells, indirect stem cell mediated beneficial effects, such as paracrine effects, anti-inflammatory activity, and immunomodulatory capacity, can also help to improve the disease. The review of Ham et al. [7] summarize the current knowledge in the field and suggests methods for treatment of OA, namely, transplantation of stem cells and differentiated MSCs using miRNA, small molecules, growth factors, and cytokines. The study of Wang et al. [8] offers further information about the articular regeneration potential, which is derived by articular MSCs injection, and supports the notion that MPCs are transplantable between HLA-incompatible individuals, a fact that could have therapeutic relevance. The indirect protective effect of MSCs, BMSCs and dermal stem cells is also demonstrated in the Special Issue for endothelial cells [9], neurogenesis [10], liver fibrosis [1,11] and skin fibroblasts [12]. These protective effects are mediated by different mechanism. The endothelial cells can be protected against glucolipotoxicity by the release of tumor necrosis factor-α stimulated protein 6 (TSG-6), as shown for human umbilical vein endothelial cells [9]. The direct and indirect stem cell dependent regenerative potential is not limited to MSCs, meanwhile several interesting adult stem cell sources, including skin, testis, ependyma and lung are recognized. Specific aspects of their regulation, differentiation and proliferation are described in this special issue [13,14,15,16]. The lung regeneration is slow, but can be improved by activation of lung progenitor cells, giving new therapeutic options, as reviewed by Akram et al. [16]. Opposite to the beneficial function of adult stem cells for maintenance and regeneration, the transformation of stem cells to tumor stem cells can drive into cancer progression. Improved knowledge about the factors, such as survivin [17], and cellular mechanisms, such as cell fusion [18], can help to develop new strategies for tumor treatment.

The broadness of the adult stem cell field is given in this Special Issue, offering several facts that can be used for a better understanding of the important role of adult stem cell activation for aging, health and diseases. Moreover, it offers an interesting background for the development of adult stem cell based therapeutic strategies.
